# Insulin-Like Growth Factor-1 Is Neuroprotective in Aged Rats With Ischemic Stroke

**DOI:** 10.3389/fnagi.2019.00349

**Published:** 2019-12-11

**Authors:** Ahmad Serhan, Erik Boddeke, Ron Kooijman

**Affiliations:** ^1^Department of Experimental Pharmacology, Center for Neurosciences (C4N), Vrije Universiteit Brussel, Brussels, Belgium; ^2^Department of Neuroscience, University Medical Center Groningen, University of Groningen, Groningen, Netherlands

**Keywords:** aging, IGF-1, stroke, microglia, neuroprotection

## Abstract

Post-stroke systemic injections of insulin-like growth factor-1 (IGF-1) exert neuroprotective effects in rats. In the current study, we aimed to test the efficacy of IGF-1 neuroprotection in aged rats (24–25 months old) and to compare the results with adult rats (6–7 months old). Furthermore, we addressed putative differences in microglial responses to IGF-1 in adult and aged rats. Rats were subjected to ischemic stroke while they were conscious by infusing endothelin-1 (Et-1) through a guide cannula that was implemented in the vicinity of the middle cerebral artery (MCA). Rats were given subcutaneous injections of IGF-1 (1 mg/kg) at 30 min and 120 min after the insult. Post-stroke IGF-1 treatment reduced the infarct size by 34% and 38% in aged and adult rats, respectively. The IGF-1 treated adult rats also showed significant improvement in sensorimotor function following stroke, while this function was not significantly affected in aged rats. Furthermore, aged rats displayed exaggerated activation of microglia in the ischemic hemisphere. Significant reduction of microglial activation by IGF-1 was only detected at specific regions in the ipsilateral hemisphere of adult rats. We show that IGF-1 reduced infarct size in aged rats with an ischemic stroke. It remains to be established, however, whether the age-related changes in microglial function affect the improvement in behavioral outcomes.

## Introduction

Ischemic stroke is one of the most common causes of death and disability (Katan and Luft, 2018). Thrombolytic therapy with recombinant tissue plasminogen activator is still the most effective therapy for ischemic stroke (Lees et al., 2016; Alberts, 2017). However, many patients are not eligible for this therapy due to the narrow therapeutic window (Lees et al., 2016; Alberts, 2017).

In stroke patients, serum levels of insulin-like growth factor-1 (IGF-1) correlate positively with clinical outcome (Åberg et al., 2011, 2018; De Smedt et al., 2011; Saber et al., 2017) suggesting that IGF-1 may exert neuroprotective effects. IGF-1 is a polypeptide hormone that is involved in neonatal and postnatal development (Agrogiannis et al., 2014; Hellström et al., 2016; de Jong et al., 2017), but also acts as a survival factor for neurons *in vitro* (Ueno et al., 2013) and *in vivo* (Wine et al., 2009).

Moreover, post-stroke treatment with systemically injected IGF-1 induced neuroprotection in several rat models for ischemic stroke (Rizk et al., 2007; De Geyter et al., 2013, 2016; Bake et al., 2014). These observations indicate that IGF-1 may be effectively used as a neuroprotective agent in patients.

Many preclinical studies successfully identified neuroprotective drugs against ischemic stroke, but these drugs failed to exert significant effects in the clinic (Green, 2008; Veltkamp and Gill, 2016). One of the recommendations of the Stroke Therapy Academic Industry Roundtable (STAIR; Fisher et al., 2009) to facilitate translation to the clinic, is to include comorbidity factors such as aging in preclinical studies. Indeed, the incidence of stroke is higher in the elderly (Béjot et al., 2016). Therefore, we tested whether IGF-1 treatment is neuroprotective in aged rats and compared the results to the efficacy of IGF-1 in adult rats. Preliminary experiments in our laboratory revealed that neuroprotection by IGF-1 in rats with ischemic stroke is accompanied by microglial changes and a decrease in neuroinflammation. Since age correlates with an exaggerated activational state of microglia (Godbout et al., 2005; Norden and Godbout, 2013), we addressed the effects of IGF-1 on microglial activation.

## Materials and Methods

Male albino Wistar Han rats were obtained from Charles River Laboratories (Germany). After transport, animals remained in the animal facility for several months under a 12-h light/dark cycle with *ad libitum* food and water. The animals were handled in accordance with the National Guidelines on Animal Experimentation and the study was approved by the Ethical Committee for Animal Experimentation of Vrije Universiteit Brussel (VUB, project number: 14-278-2).

### Surgical Operation and Induction of Stroke

Adult rats (6–7 months) were anesthetized using 1.5–3.5% isoflurane. Since aged rats (24–25 months old) were more sensitive to isoflurane gas anesthesia, they were anesthetized using 1.5–2% isoflurane. Next, the rats were fixed on a stereotactic frame and injected subcutaneously (SC) with 5 mg/kg ketoprofen. A midline incision was applied in the skull and then a burr hole was drilled carefully. Thereafter, a guide cannula (C317G/SPC, Invivo1, Roanoke, VA, USA) was inserted in the vicinity of middle cerebral artery (MCA). The coordinates for the guide cannula implementation were determined according to the Paxinos and Watson atlas (Paxinos and Watson, 2008) and the weight of the rats. The optimal coordinates for guide cannula implementation for rats weighing 275–300 g are: +0.9 mm anterior/posterior from Bregma, +5 mm lateral from Bregma and 2.8 mm ventral from dura (De Geyter et al., 2013, 2016; Zgavc et al., 2013). Since adult and aged rats were more than 450 g, the coordinates for guide cannula insertion were optimized and verified histologically afterward. For both adult and aged rats the following coordinates for guide cannula insertion were applied: +1 mm anterior/posterior from Bregma, +5.4 mm lateral from Bregma and 3 mm ventral from dura. Adult rats were left to recover for 1 day after surgery. Since aged rats appeared to be less active after surgery and more vulnerable, they were allowed to recover for 2 days. After recovery, the internal cannula (C317I/SPC, Invivo1, Roanoke, VA, USA) was connected to the guide cannula and stroke induction was induced in freely moving rats by infusing endothelin-1 (Et-1; Sigma, St. Louis, MO, USA). Et-1 is a potent vasoconstrictor which induces a 75% reduction of cerebral blood flow during 30 min after which the blood flow gradually returns to basal levels (Bogaert et al., 2000). We conducted a dose-ranging study and found that infusion of 6 μl Ringer’s solution containing 260 pmol or 120 pmol Et-1 induced comparable cerebral infarcts in adult and aged rats, respectively. The sham rats were infused with Ringer’s solution only. All rats were euthanized 24 h after induction of stroke.

### IGF-1 Injection

Our group established before that SC administration of 1 mg/kg recombinant human (rh) IGF-1 (Ipsen NV, Merelbeke, Belgium) at 30 min after stroke reduced infarct volume by 27%, but had no effect on sensorimotor function (De Geyter et al., 2013). It was also shown that increasing the dose to 3 mg/kg had no additional effect on infarct volume and did not ameliorate neurological deficits (De Geyter et al., 2013). In contrast, SC administration of IGF-1 (1 mg/kg) at 30 min and 120 min decreased the infarct volume by 32% and improved the behavioral outcome (De Geyter et al., 2013). Additional injections at 4 h and 6 h after the stroke did not lead to further ameliorations (De Geyter et al., 2013). In the current study, rats were coded and randomly assigned to vehicle or treatment groups and SC injected with a 0.4 ml solution of vehicle or IGF-1 at 30 and 120 min after the insult. Group sizes and animals excluded from the analysis are listed in Table 1.

**Table 1:** Summary of the mortality at different stages of the experiment and the reasons for exclusion with respect to estimation of infarct size and NDS.

			Adult		Aged	
	Adult	Aged	Vehicle	IGF-1	Vehicle	IGF-1
• Group size			7	9	9	12
• Excluded due to wrong guide cannula position			1	2	1	1
• Excluded due to incomplete Et-1 infusion caused by damaged internal cannula					1	3
• Excluded due to lack of good sections to stain with Cresyl Violet				2		
• Died naturally before surgery		4				
• Died during surgical procedure	2					
• Died during stroke induction	5	8				

### Sensorimotor Function

The sensory and motor functions were assessed in a blinded manner using the Neurological Deficit Score (NDS) described by Garcia et al. (1995). The NDS test was applied approximately 30 min before surgery and 24 h after induction of stroke assessing six parameters: spontaneous movement, outstretching of the forepaws, climbing, symmetrical movement of forelimb, response to vibrissae touch and body proprioception. The NDS which ranges from a minimum score of 3 to a maximum score of 18, is inversely associated with sensorimotor impairment. The NDS scores before surgery were 18 in both adult and aged rats.

### Measurement of Infarct Volume

**Figure 1:**
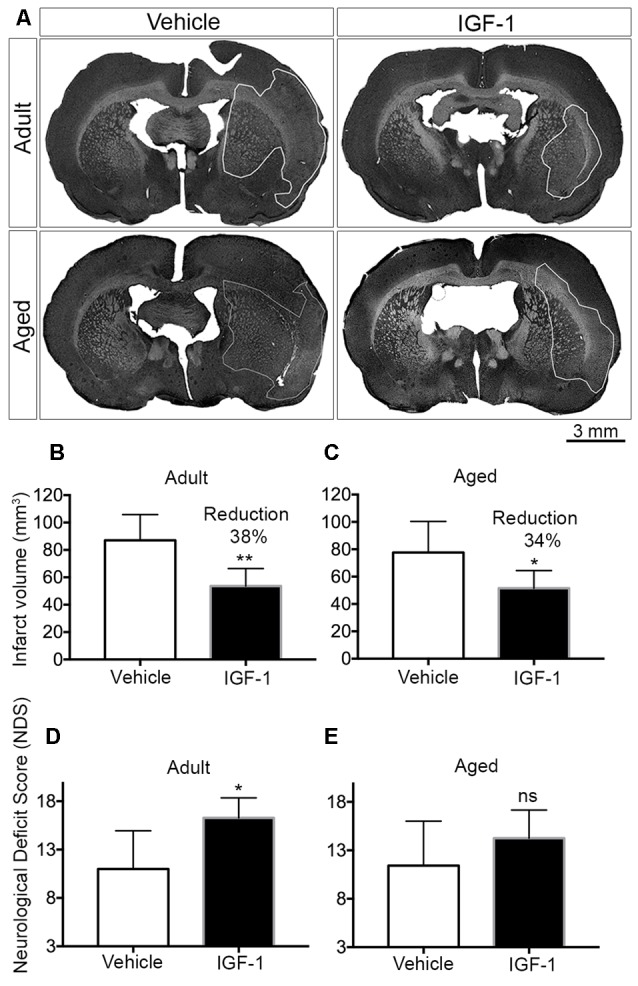
Effect of post-stroke IGF-1 treatment on infarct volume and sensorimotor function in adult and aged rats. **(A)** Representative micrographs of infarct regions in treatment and vehicle groups of both adult and aged rats. Panels **(B,C)** show the effect of IGF-1 on the infarct volume in adult and aged rats, respectively. The effect of IGF-1 treatment on sensorimotor function in adult and aged rats is represented in panels **(D,E)**, respectively. Vehicle-treated adults (*n* = 6), IGF-1 treated adults (*n* = 5 for infarct and *n* = 7 for NDS), vehicle-treated aged rats (*n* = 7) and IGF-1 treated aged rats (*n* = 8). Significance *P < 0.05; **P < 0.01. ns: not significant.

After performing the behavioral tests, rats were euthanized by intraperitoneal injection with an overdose of sodium pentobarbital. Transcardial perfusion with saline for 5 min was followed by perfusion with 4% paraformaldehyde for 5 min. Next, brains were collected and post-fixed in 4% paraformaldehyde. After post-fixation for at least 3 days, brains were cut into 50 μm coronal sections using a vibratome (Leica VTS1000, Bensheim, Germany), and stored at 4°C in phosphate buffered saline (PBS) containing 0.01% sodium azide. Assessment of infarct volume was done by mounting every fourth section on gelatin-coated slides. The slides were stained with 0.5% cresyl violet acetate and the infarcted surface areas (Figure 1A) were measured on digitized images using ImageJ software (NIH, v 1.43). The infarct volume was estimated using the following equation: v = d × Σa, where (v) is the infarct volume (mm^3^), (d) is the distance between two consecutive brain sections and (a) is the surface area of infarct size as described by Avendaño et al. (1995). Infarct volume was corrected for edema using the following equation:

Infarct volume×(volume of contralateral hemisphere/volume of ipsilateral hemisphere)

### Immunohistochemistry

**Figure 2:**
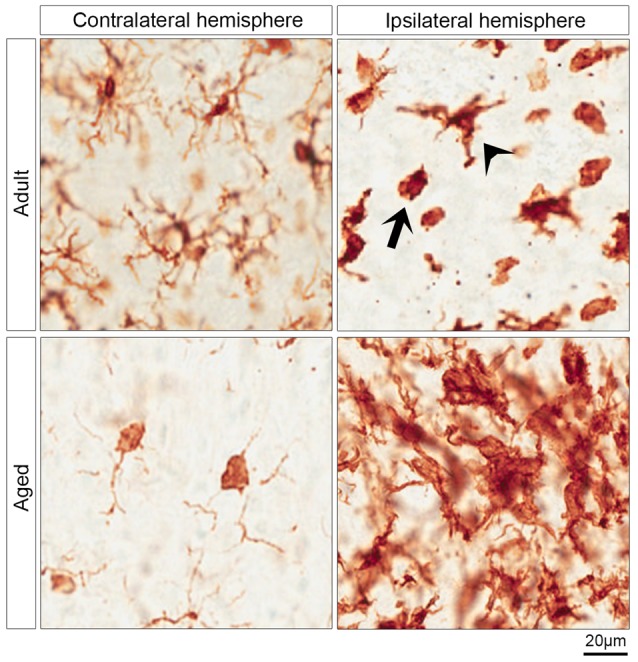
Morphology of microglia in contralateral and ipsilateral hemisphere of IGF-1 treated adult and aged rats. The upper and lower left micrographs show the surveying microglia in the contralateral hemisphere of adult and aged rats, respectively. The activated microglia are shown in the upper right micrograph which shows two morphological phenotypes of activated microglia in the striatum of the ipsilateral hemisphere of adult rats: ameboid microglia (arrow) and activated ramified microglia (arrowhead). The clusters of activated microglia in the infarcted striatum of aged rats are represented in the lower right micrograph.

**Figure 3:**
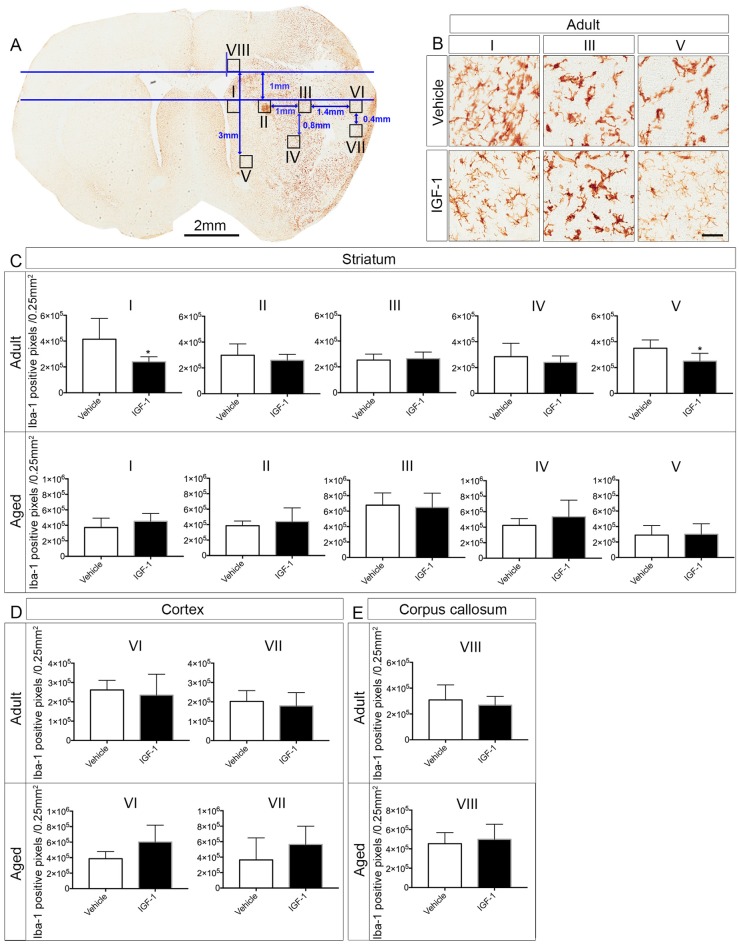
Effect of IGF-1 on the activation of microglia in adult and aged rats. **(A)** Illustration of the positions of the ROI’s in the ipsilateral hemisphere of an IGF-1 treated adult rat stained with Iba-1. **(B)** Representative micrographs of the effect of IGF-1 on the activation of microglia in regions I, III and V of the striatum. Panels **(C–E)** display the differences in Iba-1 positive pixels (number of pixels/0.25 mm^2^) in different regions in the striatum, cortex and corpus callosum in adult and aged rats. Scale bar of the micrographs in **(B)** is 50 μm. The number of adult rats per each group: All regions for vehicle and IGF-1 treatments (*n* = 5), except for vehicle region VI and IGF-1 region VII (*n* = 4). The number of aged rats per each group: Vehicle and IGF-1 regions I, II, III, IV and V (*n* = 7), vehicle region VI (*n* = 4), vehicle and IGF-1 region VII, IGF-1 region VI and vehicle region VIII (*n* = 6) and IGF-1 region VIII (*n* = 5). Significance *P < 0.05.

Two or three coronal brain sections (50 μm) were taken to assess the microglia activation using ionized calcium binding adaptor molecule-1 (Iba-1) staining. The brain sections were selected as follows: one brain section near 0.90 mm anterior to Bregma, one brain section near 0.26 mm posterior to Bregma and one brain section in the middle of the aforementioned coordinates. After subsequent incubation in 3% H_2_O_2_ in water (30 min), 0.1% Triton X-100 in water (15 min) and 20% normal goat serum in PBS (30 min), brain sections were stained overnight with rabbit anti-Iba-1 (1:1,000 dilution in 20% normal goat serum in PBS; Wako, Japan) at 4°C. Next, brain slices were incubated with horseradish peroxidase-conjugated donkey anti-rabbit IgG (NA934V, GE Healthcare, UK; 1:100 dilution in 20% normal goat serum in PBS) for 4 h at room temperature and binding of this secondary antibody was visualized using the diaminobenzidine substrate chromogen kit (Dako Cytomation, Glostrup, Denmark). Negative controls were incubated in normal goat serum without the primary antibody. The brain slices were cover-slipped using DPX mounting medium and scanned afterward with Aperio CS2 scanner (Leica, Belgium). The brain sections that are stained with Iba-1 in adult and aged rats are shown in Figure 2. The regions of interests (ROI’s; 0.25 mm^2^ each) to study microglial activation in the ipsilateral hemisphere were selected using the ruler tool in Aperio ImageScope software (Version 12.1.0.5029, Leica, Belgium). The ROI’s were selected in the infarcted regions (II, III and IV in the striatum and VI and VII in the cortex), at the border of the infarct (I and V) or outside the infarct (region VIII in the corpus callosum) using adult vehicle-treated rats. A horizontal line was annotated on top of the striatum of the two hemispheres and this line was used as a reference to determine the position of the ROI’s on the *Y*-axis (Figure 3A). Then, a parallel line was drawn and regions I and III were annotated along this line at the edges of the striatum. Subsequently, regions II, IV and VI were annotated by taking region III as a reference and region VII was selected based on region VI. A vertical line crossing the top of the corpus callosum in the ipsilateral hemisphere was used as a reference to annotate region VIII in the corpus callosum. Region V was selected based on region VIII as indicated in Figure 3A. After annotating the ROI’s, microglial activation was assessed by quantifying the number of Iba-1 positive pixels which were analyzed by an Aperio Positive Pixel Count v9 algorithm. Three categories of pixels (weak positive, positive and strongly positive) were generated, summed and quantified as the number of positive pixels/0.25 mm^2^.

### Statistics

All data are presented as mean ± SD. Statistical significance for the infarct volume and Iba-1 positive pixels between vehicle- and IGF-1-treated groups were analyzed using the unpaired student’s *t*-test. The NDS score was analyzed by the Mann–Whitney test. The results were considered significant when *P* < 0.05. Statistical analysis was performed using GraphPad Prism 7 (version 7.0a for MacOS, GraphPad Software, La Jolla, CA, USA).

## Results

### Effect of Post-stroke IGF-1 Administration on Infarct Size and Sensorimotor Function

Subcutaneous injections of IGF-1 at 30 min and 120 min after the insult resulted in a significant reduction of infarct volume by 38% and 34% in adult and aged rats, respectively (Figures 1A–C). A concomitant improvement in sensorimotor behavior was observed in both adult and aged rats, but this effect only reached significance in adult rats (Figures 1D,E).

### Modulation of Microglial Activation by IGF-1 in Adult and Aged Rats

Although microglia and macrophages share morphological features and express similar markers including Iba-1, we assessed the activation of microglia by Iba-1 staining as macrophages do not infiltrate the brain within 24 h of stroke onset (Schilling et al., 2003). The surveying microglia are found in the contralateral hemisphere, while the activated microglia are predominantly abundant in the ipsilateral hemisphere (Figure 2). The activated microglia in adult rats were morphologically classified into activated ramified and ameboid microglia. The former exhibit a dense cell body and thick ramifications (Figure 2, arrowhead), while the latter are characterized by a round-shaped and dense cell body, and phagocytic activity (Figure 2, arrow). Furthermore, Figure 2 reveals that surveying microglia in the contralateral hemisphere of aged rats exhibited a different morphological phenotype from that of adult rats. Surveying microglia in the striatum of aged rats had small or no ramifications compared to the surveying microglia in adult rats (Figure 2). On the other hand, aged rats displayed aggregates of activated microglia in the ipsilateral hemisphere of rats with an ischemic stroke (Figure 2).

To address the effect of IGF-1 treatment on microglial activation in the ipsilateral hemisphere, we analyzed Iba-1 positive pixels in ROI’s that were selected from the infarcted and non-infarcted regions in the vehicle-treated adult rats. It appeared that IGF-1 decreased the number of Iba-1 positive pixels at the border of infarction in the striatal regions I and V of adult rats, but was without effect in other regions in the striatum, cortex and corpus callosum (II, III, IV, VI, VII and VIII; see Figures 3B–E). In contrast to adult rats, aged rats did not show a reduction in positive pixels in the striatal regions I and V (Figure 3C). Furthermore, the microglial activation in other regions was also not affected by IGF-1 treatment of aged rats (Figures 3B–E).

## Discussion

IGF-1 has been used extensively as a potent neuroprotective agent in several animal models for ischemic stroke (Guan et al., 2001; De Geyter et al., 2013; Bake et al., 2014). However, comorbidities may affect the treatment efficacy in preclinical models, e.g., the neuroprotective effect of IGF-1 following ischemic stroke was impaired in hypertensive rats, compared to normotensive rats (De Geyter et al., 2013). It has also been shown that post-stroke treatment with fluoxetine, a selective serotonin reuptake inhibitor, was neuroprotective and improved the sensorimotor function in adult rats (Lim et al., 2009), while fluoxetine treatment following stroke in aged rats did not have an effect on infarct volume and behavioral outcome (Zhao et al., 2005). Also, the beneficial effects of leukemia inhibitory factor (Davis et al., 2019) and apocynin (Kelly et al., 2009) which were observed in young adult rats were absent in aged rats.

Age is one of the most prominent risk factors for stroke and can be modeled in rodents. We addressed the potential neuroprotective effects of systemic administration of IGF-1 in aged Wistar Han rats using the Et-1 model for the induction of ischemic stroke in conscious animals. We previously showed, using this model, that SC injection of IGF-1 following stroke reduced infarct volume by 32% in 3-month-old rats and improved the NDS score (De Geyter et al., 2013). In the current study, we show similar results in 6–7 month-old rats in which IGF-1 reduced infarct volume by 38% and compared the neuroprotective effects of IGF-1 in these rats with the effects in 24–25-month-old rats (Figures 1A–C). To our knowledge, we are the first to demonstrate that post-stroke systemic administration of IGF-1 reduces infarct size in aged rats (Figures 1A,C). Although IGF-1 treatment significantly improved the NDS following ischemic stroke in adult rats (Figure 1D), this score was not significantly improved in aged rats (Figure 1E). The smaller effect on sensorimotor behavior may be due to the finding of DiNapoli et al. (2008), that there is a delay in recovery after ischemic stroke in older animals or may be related to the exaggerated activation of microglia in the ipsilateral hemisphere (Figure 2). Another explanation could be that in aged rats a further reduction in infarct volume may be needed to achieve a significant improvement in sensorimotor functions. The latter explanation is in line with our observation that a 32% reduction in infarct size coincides with an improvement in behavioral outcome, while a 27% reduction was without effect (De Geyter et al., 2013). Moreover, future studies, with larger sample sizes, are recommended to establish the effect of IGF-1 at different time points beyond 24 h of stroke induction, especially with respect to possible short-term confounding effects of the anesthesia before the induction of stroke. Alternatively, adjustments of the dose of IGF-1 may be required to significantly augment sensorimotor behavior in aged rats. Although the NDS test is easy to conduct on animals, it is only suitable for testing during the acute phase of ischemic stroke, as the assessed neurological deficits disappear within a few days after stroke (Balkaya et al., 2018). Hence, long-term studies using alternative behavioral tests are warranted.

Although one of the advantages of the Et-1 model is that stroke is induced in conscious animals in the absence of neuroprotective anesthetics, the possibility remains that the outcome will be influenced by the long-term effects of anesthesia. In addition, these long-term effects may be different in aged rats compared to adult rats, also, because of the differences in doses of isoflurane that the animals had been exposed to. In future studies, these differences can be minimized by allowing the rats to recover for more than 2 days after surgery. Nevertheless, a limitation of our study with respect to the comparison between adult and aged rats is that aged rats were more vulnerable than adult rats and required adaptations in the procedures for surgery and induction of stroke by Et-1.

To assess whether activation of microglia is instrumental to neuroprotection, microglia were stained with Iba-1. The neuroprotective effect of IGF-1 in adult rats following ischemic stroke was paralleled by a decrease in Iba-1 positive pixels in regions I and V at the border of the infarct (Figures 3A–C). The reduction on microglial activation in regions I and V which are, at least partially, salvaged by IGF-1 treatment in adult rats (Figure 1A) is to be expected, but the absence of this reduction in aged rats is remarkable. It could be that alleviating the activation of microglia at the border or in the vicinity of the infarcted regions may be necessary for the improvement in functional outcome. Our observation that IGF-1 does not affect Iba-1-staining in aged rats could be due to age-dependent alterations in the activational state of microglia. Indeed, surveying microglia in aged rats showed relatively small or no protrusions and dense soma in the contralateral hemisphere (Figure 2). This is in agreement with another study that demonstrated microglia in aged mice have shorter ramifications, enlarged cell body and non-homogenous distribution (Damani et al., 2011; Hefendehl et al., 2014). Moreover, it has been shown that microglia in the aged brain are primed (or reactive), have pro-inflammatory profile and are characterized by an increased level of activation markers including CD86, major histocompatibility complex, toll-like receptors (Frank et al., 2006; Letiembre et al., 2007; Norden and Godbout, 2013). It has also been shown that aged mice display an increased and prolonged secretion of pro-inflammatory cytokines in response to lipopolysaccharide, compared to adult mice (Norden and Godbout, 2013). Our finding that aged rats have clusters of activated microglia in the ipsilateral hemisphere of the ischemic brain (Figure 2), is also consistent with these observations in the literature. Since it is not possible to characterize the different types of microglia in aged rats using 2D-images (Figure 2), the use of confocal stack images for 3D cell reconstruction employed by Wagner et al. (2013), could be useful in future studies on the role of glial cells in neuroprotection in aged rats.

Another limitation of the Et-1 model is that an invasive method is required to infuse Et-1 next to the MCA. Indeed, in aged rats, the insertion of the guide cannula evoked activation of microglia in the ipsilateral hemisphere as assessed by Iba-1 staining in sham rats. Although establishing the effects of IGF-1 on these artifacts in sham rats would be worthwhile, the effects of IGF-1 on microglial activation in regions I and V shown in Figure 3, were located outside the region that was affected by the insertion of the guide cannula. Although reactive microglia may adversely affect the efficacy of treatment due to their exaggerated inflammatory responses, our results suggest that the increase in reactive microglia following stroke did not affect the reduction in infarct size in aged rats.

Taken together, our results clearly demonstrate that IGF-1 exerts a promising neuroprotective effect in aged rats when it comes to the reduction in infarct size. However, it remains to be established whether age in stroke patients could influence the clinical outcome after IGF-1 treatment. Future studies are warranted to shed light on the underlying mechanisms of IGF-1 neuroprotection in ischemic aged rats and the role of the microglia.

## Data Availability Statement

The raw data supporting the conclusions of this article will be made available by the authors, without undue reservation, to any qualified researcher.

## Ethics Statement

The animals were handled in accordance with the National Guidelines on Animal Experimentation and the study was approved by the Ethical Committee for Animal Experimentation of Vrije Universiteit Brussel (VUB).

## Author Contributions

RK designed the experiments. AS conducted the experiments, analyzed the data and wrote the manuscript. RK and EB supervised the work and revised the manuscript. All authors read and approved the submitted version of the manuscript.

## Conflict of Interest

The authors declare that the research was conducted in the absence of any commercial or financial relationships that could be construed as a potential conflict of interest.
